# Nurse-Led Interventions and Outcomes in Adult Hematopoietic Stem Cell Transplant Recipients: A Scoping Review

**DOI:** 10.3390/nursrep16090344

**Published:** 2026-09-20

**Authors:** Joshua Kanaabi Muliira, Eilean Rathinasamy Lazarus

**Affiliations:** 1 Department of Adult Health & Critical Care, College of Nursing, Sultan Qaboos University, Muscat 123, Oman; j.muliira@squ.edu.om; 2 Medical Research Center, Sultan Qaboos University, Muscat 123, Oman

**Keywords:** hematopoietic stem cell transplantation, nurse-led intervention, quality of life, self-management, bone marrow transplantation

## Abstract

**Background/Objectives:** Hematopoietic cell transplantation (HCT) is increasingly used to treat malignant and non-malignant hematologic diseases and is associated with complex physical, psychological, and informational needs. Nurses play a central role in HCT care; however, the range, characteristics, and reported outcomes of nurse-led interventions have not been comprehensively synthesized. This review aimed to identify the types and outcomes of nurse-led interventions in adult HCT recipients and highlight gaps in evidence. **Methods:** The review was initially registered as a systematic review and was refined to a scoping review before final synthesis because of substantial heterogeneity in the available evidence. Studies involving adults undergoing or having received HCT were included if they evaluated interventions initiated, delivered, or coordinated by nurses and reported at least one clinical or patient-reported outcome. Multiple databases and grey literature (2015–2025) were searched. Study selection and data charting were performed independently by two reviewers. Methodological quality was assessed using the Mixed Methods Appraisal Tool, and findings were synthesized narratively. **Results:** Ten studies met the inclusion criteria, mostly from high-income countries. Nurse-led interventions included pre-transplant preparation, education, symptom management, physical activity and nutrition support, psychosocial care, navigation, survivorship follow-up, and advance care planning. Several studies reported improvements in self-efficacy, physical activity, symptom burden, and advance care planning documentation; however, findings for overall quality of life, infection rates, and long-term recovery were limited or inconclusive. **Conclusions:** Evidence on nurse-led interventions across the HCT trajectory remains limited. The mapped studies provide promising preliminary findings in selected outcome domains but are insufficient to establish intervention effectiveness. Future research should develop and evaluate rigorously designed, adequately powered, longitudinal, and multicomponent nurse-led interventions, including in resource-limited settings.

## 1. Introduction

Hematopoietic cell transplantation (HCT) is an essential therapy for many malignant and nonmalignant hematologic diseases [1]. HCT refers to a procedure where hematopoietic stem cells (HSCs) of any donor type and source are given to a recipient with the intention of repopulating and replacing the hematopoietic system in total or in part [2]. HSCs for HCT can be derived from bone marrow, peripheral blood, or cord blood [2]. Due to the increasing expansion of HCT technology, such as unrelated donor and haploidentical HCT, use of reduced intensity HCT in older patients, and the rise in cellular therapy, there is a global increase in the number of patients undergoing HCT [3], and subsequently, a demand for evidence-based short-term and long-term nursing care for recipients.

The use of HCT is increasing globally [4,5]. For instance, from 2009 to 2016, the number of HCTs used in the treatment of Acute Myeloid Leukemia increased by 54.9% globally [5]. The biggest increase was in high-income countries, but also large increments were registered in resource-constrained regions such as Africa and the East Mediterranean Region (94.6%) and the America-Nord Region (34.7%) [5]. HCT is used in the management of other malignant conditions such as Acute Myeloid Leukemia, Acute Lymphoblastic Leukemia, Myeloproliferative Neoplasms, Chronic Myelomonocytic Leukemia, Chronic Lymphocytic Leukemia, T-cell Lymphoma, multiple myeloma, and others [2].

HCT therapies also play a critical role in the management of acquired severe aplastic anemia, amyloidosis, inherited hemoglobinopathies, and inborn errors of metabolism [2]. Increasingly, HCT therapies are being used in the management of autoimmune disorders such as multiple sclerosis, systemic sclerosis, Crohn’s disease, systemic lupus erythematosus, neuromyelitis optica, chronic inflammatory demyelinating polyradiculoneuropathy, myasthenia gravis, stiff person syndrome, systemic vasculitis, Takayasu, Behçet’s disease, refractory coeliac disease, and others [2]. Despite the increased utilization, there are still several challenges that limit the use and outcomes of HCT therapies. HCT is one of the most complex forms of treatment because it involves intensive conditioning therapy, infusion of hematopoietic cells, engraftment and immune reconstitution, and close monitoring for treatment-related toxicities and infections [6]. In allogeneic HCT, the need to prevent, identify, and manage acute and chronic graft-versus-host disease further increases clinical complexity. Recipients may also experience multisystem physical, psychological, and social consequences during the acute treatment phase and throughout long-term survivorship, requiring coordinated multidisciplinary care and follow-up [6].

Other challenges include persistent disparities in utilization of the procedure and poor outcomes in racial and ethnic minority patients [7]. Moreover, patients undergoing HCT and their family caregivers experience a high level of uncertainty and distress, and low levels of preparedness [8], indicating a gap in nursing care and healthcare services in general. The other challenges associated with HCT include post-HCT complications such as graft-versus-host disease (GvHD), oral mucositis (OM), sepsis [6], and others. Even one-year post-HCT, patients continue to report a high symptom burden with problems such as severe fatigue, susceptibility to infection, sexual dysfunction, and poor general health [9], and these impact quality of life and other outcomes.

However, the challenges and process of caring for HCT recipients offer a wealth of opportunities for nurses to make a positive difference to the patient experience through high-quality specialist evidence-based nursing care, innovative nurse-led interventions, and the execution of unique roles within the transplant therapy pathway [6]. For instance, to guarantee good outcomes among HCT recipients, nurses can intervene to ensure that patients receive quality nursing care and management during hospitalization and have a deeper understanding of their condition and therapy [10]. Moreover, during the transplant care trajectory, nurses can utilize evidence-based nurse-led interventions as they serve in key roles such as transplant coordinator, apheresis nursing care, ambulatory and day unit nursing care, and follow-up care [6].

Nursing care during the HCT care trajectory can also utilize innovative models to manage symptoms, educate patients on ways to self-manage multiple symptoms, and train HCT recipients in self-management skills to enhance quality of life [10]. The above nursing roles, along with the care required by HCT recipients during pre-transplantation and post-transplantation and the protracted impact of the symptom burden, suggest that nursing is a critical part of the recovery trajectory. Therefore, nurse-led interventions can help HCT recipients cope with the associated challenges, such as the longer recovery process, long-term physical symptoms, survivorship uncertainty, social stigma and discrimination related to being perceived as a burden, self-management skills, and others. These interventions may address important supportive-care needs across the HCT trajectory; however, their impact on patient and health-system outcomes requires more rigorous evaluation. However, there has been a lack of a clear synthesis of nurse-led interventions and their impact on HCT recipients’ outcomes. This gap in knowledge limits the development and rigorous evaluation of nurse-led interventions tailored to the needs of HCT recipients. The review aimed to identify the types and outcomes of nurse-led interventions used in adult recipients of HCT. The findings identify gaps in the available evidence and areas for future development and evaluation of nurse-led interventions that may support nursing care, patient outcomes, and health-system processes. These intervention areas warrant further development and rigorous evaluation to determine their feasibility, acceptability, and potential contribution to patient and health-service outcomes.

## 2. Methods

This scoping review aims to map and synthesize global evidence about nurse-led interventions that influence health outcomes among adult HCT recipients. The review also describes the intervention characteristics, contexts, outcomes, and gaps with respect to transplant nursing practice.

### 2.1. Methodological Framework

This scoping review was conducted using the framework proposed by Arksey and O’Malley, refined by Levac et al., [11] and informed by Joanna Briggs Institute (JBI) guidance for scoping reviews [12]. Reporting followed the Preferred Reporting Items for Systematic Reviews and Meta-Analyses Extension for Scoping Reviews (PRISMA-ScR) (PRISMA-ScR Checklist Supplementary File S1) [13,14]. Given the expected heterogeneity in populations, intervention types, comparators, and outcome measures, findings were synthesized narratively rather than statistically pooled, consistent with the purpose of a scoping review. As meta-analysis was not appropriate, principles from the Synthesis Without Meta-analysis (SWiM) reporting guideline were used solely to improve transparency in how studies were grouped and summarized in the narrative synthesis [14,15]. The protocol was registered prospectively in PROSPERO (CRD420251273588), and no amendments were made after registration.

### 2.2. Identifying the Research Question

The review team comprised senior oncology nurses, academics, bone marrow transplant nurse specialists, researchers experienced in evidence synthesis, and a health sciences librarian, reflecting the multidisciplinary composition recommended by JBI for scoping reviews and commonly used in complex oncology topics. Through iterative meetings and application of a Population–Concept–Context (PCC) framework [16] (Kim et al., 2025), the team agreed on the following overarching questions, consistent with the revised aim: (1) What types of nurse-led interventions have been evaluated among adult patients undergoing hematopoietic stem cell/bone marrow transplantation across the pre-transplant, inpatient, and post-transplant continuum, and what outcomes have been reported?; and (2) which health outcomes (e.g., symptom burden, treatment-related complications, psychological well-being, functional status, health-related quality of life, survival, and healthcare utilization) are targeted by nurse-led interventions? These questions align with the aims of scoping reviews to map the breadth, range, and nature of existing research and to highlight gaps to guide future studies and practice.

### 2.3. Key Concept Definitions

To ensure conceptual clarity and consistency, this review adopted working definitions for its three central concepts: hematopoietic stem cell/bone marrow transplantation, nurse-led intervention, and patient self-management/supportive care. HCT/bone marrow transplantation (HCT/BMT) is defined as the intravenous infusion of autologous or allogeneic hematopoietic stem and progenitor cells to re-establish bone marrow function in patients with malignant or non-malignant hematologic and immune disorders, typically delivered within a trajectory that includes pre-transplant conditioning, the inpatient transplant admission, and early post-discharge recovery [17]. This definition reflects contemporary HCT practice across indications such as leukemia, lymphoma, multiple myeloma, and selected non-malignant diseases.

Nurse-led intervention refers to an evidence-based, person-centered model of care in which registered nurses or advanced practice nurses hold primary responsibility for assessment, delivery, coordination, and follow-up of the intervention, exercising meaningful autonomy in clinical decision making while often working within multidisciplinary teams [18,19]. In the HCT/BMT context, this includes nurse-delivered education classes, navigation and follow-up services, symptom-management and self-care coaching, psychosocial or relaxation interventions, and embedded quality-improvement protocols in routine transplant care.

Self-management and supportive care are defined as the patient’s (and caregiver’s) ability, supported by health professionals, to manage symptoms, treatments, physical and psychosocial consequences, and lifestyle changes associated with cancer and HCT/BMT across the care continuum [20]. In this review, self-management encompasses medical management (e.g., infection prevention, medication adherence, nutrition, and physical activity), role management (maintenance or adaptation of family and social roles), and emotional management (coping with distress, uncertainty, and fatigue), with nurses providing structured education, coaching, and follow-up to enable these behaviors during and after transplantation [21,22].

### 2.4. Identifying Relevant Studies

Consistent with PRISMA-ScR and JBI guidance, a comprehensive search strategy was developed in close collaboration with a senior health sciences librarian, using both controlled vocabulary (e.g., MeSH, Emtree, CINAHL Headings) and free-text terms for three core concepts: hematopoietic stem cell/bone marrow transplantation, nurse-led or nursing interventions, and patient health outcomes. The following databases were searched from 1 January 2015 to 31 December 2025: MEDLINE (Ovid), EMBASE (Ovid or Embase.com), CINAHL (EBSCOhost), Scopus (Elsevier), Web of Science Core Collection (Clarivate), and PsycINFO (Ovid). The 2015–2025 period was selected to focus the review on contemporary nurse-led interventions delivered within current HCT practice. This period reflects major developments in transplant care, including evolving donor and conditioning approaches, increasing outpatient and survivorship care, greater emphasis on patient-reported outcomes and self-management, and the emergence of digital approaches to supportive care. The date restriction also ensured that the review remained feasible while mapping interventions most relevant to current clinical contexts.

### 2.5. Search Strategy

Keywords for the three main concepts were grouped as follows. For the transplant population, the terms included bone marrow transplant, hematopoietic stem cell transplant, HCT, hematopoietic cell transplant, stem cell transplant, autologous transplant, and allogeneic transplant. For nurse-led interventions, the terms included nurse-led, nursing intervention, nurse-managed, nurse-delivered, clinical nurse specialist, transplant nurse, oncology nurse, advanced practice nurse, and nurse navigator. For outcomes, the terms included symptom, symptom burden, pain, fatigue, psychological distress, anxiety, depression, self-care, self-management, quality of life, health-related quality of life, patient-reported outcomes, treatment-related complications, infections, graft-versus-host disease, functional status, treatment adherence, medication adherence, survival, readmission, and healthcare utilization. Complete, database-specific search strategies, limits, and record counts for each source are provided in Supplementary File S2, including line-by-line search terms (Table 1) for EMBASE, CINAHL, Scopus, Web of Science Core Collection, ScienceDirect, ProQuest, and OpenGrey and the exact date each database was searched. The main database searches were conducted between 13 and 14 December 2025, with additional updated runs on 5 January 2026, as detailed in Supplementary File S2 (Supplemental File S2—Detailed Database Search Strategy).

### 2.6. Selecting Studies

**Figure 1:**
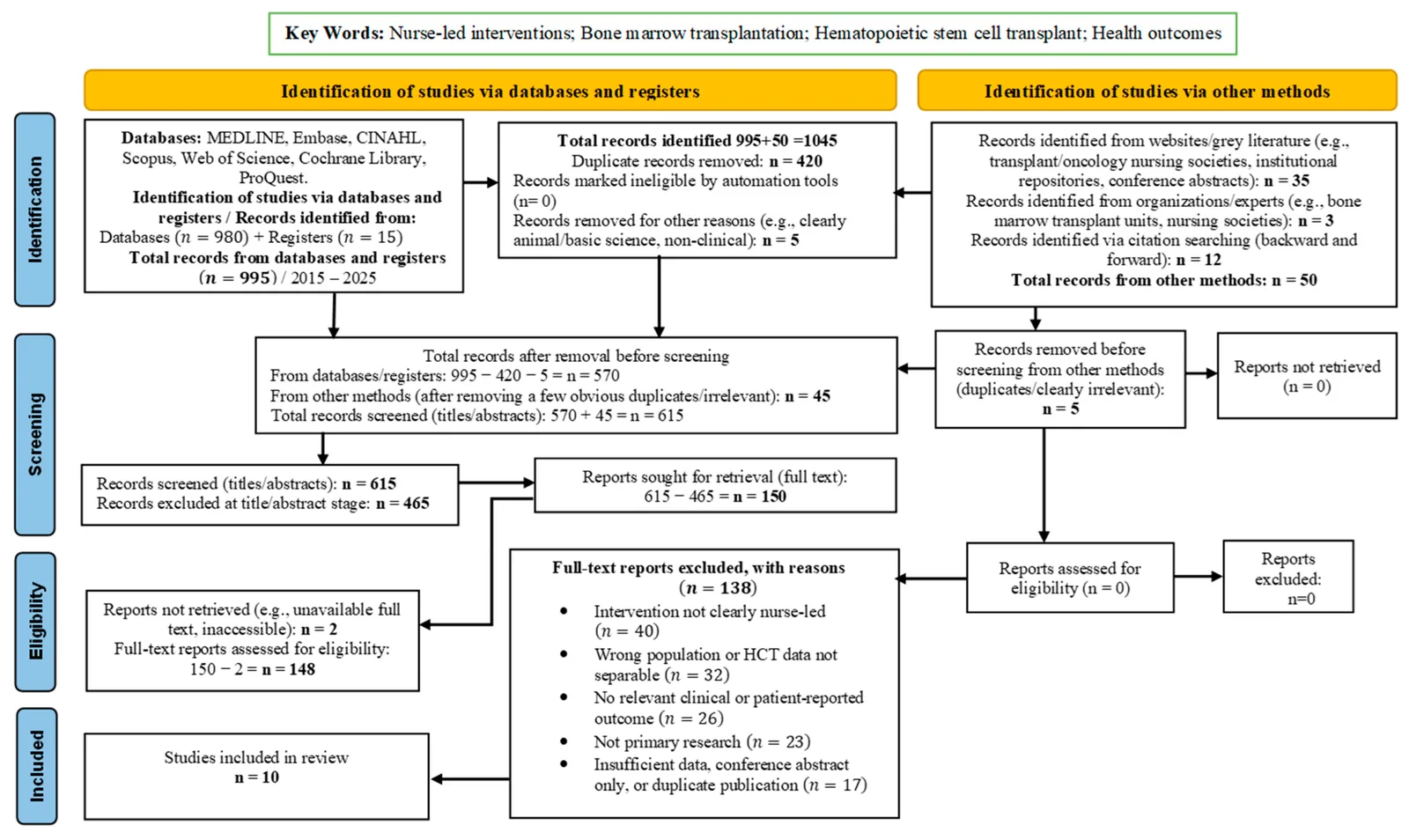
PRISMA-ScR flow diagram of study selection [23].

All search results were exported in RIS/BibTeX format and combined into a single EndNote 21 library for reference management and deduplication. Automatic duplicate detection in EndNote was applied first, followed by manual checking of near-duplicates (e.g., small variations in titles, author names, or journal details) by members of the review team (JKM and ERL) to ensure each unique study was represented once before screening. The dataset was then imported into a web-based screening platform Rayyan tool, where two reviewers independently screened titles and abstracts against predefined eligibility criteria; records deemed potentially relevant by either reviewer progressed to full-text assessment. Full-text screening was conducted in duplicate by the same pairs of reviewers, with disagreements at any stage resolved through discussion, and, when needed, adjudication by a third reviewer (PN) from the team. The overall study selection process, including reasons for full-text exclusion, is summarized in a PRISMA 2020-aligned flow diagram (Figure 1) adapted for scoping reviews.

### 2.7. Inclusion and Exclusion Criteria

The eligibility criteria were defined a priori using the Population–Concept–Context framework recommended by JBI for scoping reviews. The inclusion criteria were defined using the Population–Concept–Context framework. The population comprised adult patients undergoing hematopoietic stem cell/bone marrow transplantation at any phase of the care continuum, including pre-transplant preparation, inpatient transplant admission, early post-transplant recovery, longer-term survivorship, and late-effects follow-up. The concept focused on nurse-led interventions, i.e., interventions initiated, delivered, or primarily coordinated by registered nurses, clinical nurse specialists, nurse navigators, or advanced practice nurses in transplant, hematology, or oncology settings, delivered via in-person, telephone, or digital/telehealth modalities. The context included any healthcare or community setting (such as transplant units, hematology/oncology wards, outpatient transplant clinics, survivorship or late-effects clinics, and community or home-based programs) in any country or healthcare system. Eligible study designs were primary empirical quantitative studies (e.g., randomized or non-randomized trials, cohort, and pre–post designs), qualitative studies, and mixed-methods studies that described or evaluated nurse-led interventions and reported at least one relevant health outcome, including symptom-related, complication-related, psychological, functional, quality-of-life, survival, or healthcare-utilization outcomes, and published between 2015 to 2025. No language limits were applied at the search stage. During screening, however, inclusion was restricted to full-text articles available in English. This pragmatic decision reflected the review team’s limited capacity for translation and aimed to minimize misinterpretation of complex clinical and intervention details.

Studies were excluded if the intervention was not clearly nurse-led (for example, multidisciplinary or physician-led programs without a definable nursing lead), if the population did not include transplant recipients or data for patients receiving hematopoietic stem cell/bone marrow transplantation could not be separated from other groups, or if the focus was exclusively on donor management, screening, or primary prevention rather than post-transplant care. Non-empirical publications such as protocols, narrative or systematic reviews, commentaries, editorials, letters without primary data, and abstract-only conference reports with insufficient detail for charting were also excluded. Where multiple reports described the same intervention cohort, they were collated and treated as a single study in the synthesis. Where a multi-arm study included both nurse-led and non-nurse-led interventions, only data from the arm meeting the predefined nurse-led intervention criteria were included in the narrative synthesis.

### 2.8. Data Extraction

The selection process is displayed using a PRISMA-ScR-informed flow diagram to provide a transparent account of record identification, screening, eligibility assessment, and inclusion. A standardized data-charting form was constructed in line with JBI and PRISMA-ScR guidance and piloted on a subset of included studies, after which minor refinements were made to improve clarity and consistency before full data extraction. Two reviewers (JKM and ERL) independently charted data from each of the included studies into the form and then compared entries; any discrepancies were resolved by discussion, and when needed, with input from a third team member (PN).

Data items extracted included study characteristics (first author, year of publication, country, setting, study aims, and design) and participant characteristics (sample size per arm, mean or median age). Details of the interventions were charted, including intervention name or brief description, core components, and delivery mode (e.g., relaxation or healing-touch interventions). Contextual information captured included the country and setting (e.g., university hospital transplant unit, hematology department, comprehensive cancer center, outpatient clinic). The data about the outcomes of the intervention were extracted and this included variables such as symptom burden, infection rate, length of stay in the hospital, psychological distress, quality of life, and others. Key findings relevant to the research questions were summarized, including any reported barriers and facilitators with respect to implementing nurse-led interventions within transplant settings whenever available.

### 2.9. Collating, Summarizing, and Quality Assessment

Given the expected heterogeneity in populations, intervention types, comparators, and outcome measures, findings were synthesized narratively rather than statistically pooled, consistent with the purpose of a scoping review. Studies were grouped thematically by intervention type, including symptom-management and supportive-care program, psychoeducational or counselling interventions, survivorship and late-effects follow-up models, and care-coordination or navigation programs. Studies were also grouped by outcome domains: quality of care, symptom burden and complications, psychological outcomes, quality of life and functioning, and self-management. The review was reported in accordance with the Preferred Reporting Items for PRISMA-ScR. As meta-analysis was not appropriate, principles from the SWiM guideline were used solely to improve transparency in how studies were grouped and summarized in the narrative synthesis. The synthesis was intended to map intervention characteristics and patterns in reported outcomes; it was not intended to estimate pooled effects, compare interventions, or establish causal effectiveness.

Consistent with the exploratory purpose of this scoping review, no formal design-specific risk-of-bias assessment was undertaken. Instead, a descriptive methodological appraisal was conducted using the MMAT, version 2018 [24]. Each included study was first classified according to its methodological design, and the corresponding MMAT criteria were applied. Randomized controlled trials were assessed for the appropriateness of randomization, baseline comparability, completeness of outcome data, blinding of outcome assessors, and adherence to the assigned intervention. Non-randomized studies were assessed for participant representativeness, appropriateness of outcome and intervention measurements, completeness of outcome data, consideration of confounding, and whether the intervention was delivered as intended. For mixed-methods studies, the quantitative and qualitative components were assessed using the relevant MMAT criteria, followed by appraisal of the rationale for using mixed methods, integration and interpretation of findings, consideration of inconsistencies between components, and the quality of each component. Appraisal findings were used descriptively to identify recurring methodological strengths and limitations and to contextualize the evidence map; they were not used to exclude studies, calculate overall quality scores, or weight the narrative synthesis. Study classifications, MMAT categories, and key methodological observations are presented in Table 2.

Data were charted using a structured form developed and pilot-tested by the review team in line with Arksey and O’Malley, Levac et al., and JBI guidance for scoping reviews. The form captured bibliographic information, country and setting, study design, participant characteristics, transplant type and phase, detailed characteristics of the nurse-led intervention (content, theoretical underpinning, mode of delivery, duration and intensity, and provider role), comparators where applicable, outcome domains and instruments, follow-up period, and key findings, including feasibility and implementation information. Two reviewers independently charted data, with discrepancies resolved through discussion or consultation with a third reviewer.

### 2.10. Ethical Considerations

This scoping review used only publicly available published literature and no identifiable data. Therefore, ethics committee approval was not required. All sources were cited and reported transparently.

## 3. Results

### 3.1. Characteristics of the Studies

Of the 1045 articles found in the search databases, ten (10) were selected for inclusion in the scoping review. The results presented in Table 3 show that most of the nurse-led intervention studies (3) used quasi-experimental designs [29,31,32] and randomized controlled trial designs (3) [25,26,27]. The other designs were single-group intervention studies [33,34], a mixed-methods design [30], and a quality improvement intervention project [28]. Two of the studies were from reports obtained from graduate nursing students’ theses [28,34], highlighting the contribution of advanced nursing education on knowledge development. One study focused on both the nurses and patients [31], and all the remaining nine studies had samples comprised of HCT recipients. The mean age of the patients was in the range of 41 to 58 years, and the patients with the lowest mean age were in a sample from China [32].

Most studies (4) were conducted in the USA [25,26,28,31] and the others were from Sweden [29,34], the Netherlands [30], China [32], Turkey [27], and Japan [33]. Therefore, most studies were conducted in high-income countries in North America, Europe, and one middle-to-high-income country in Asia. There were no studies from low-to-middle-income countries (LMICs) of Asia, Africa, and South America, despite the increased use of HCT in these settings. These gaps in nursing scientific literature limit our understanding of HCT-related nursing care in these settings, where low-cost and effective interventions are needed due to the escalating cancer burden.

### 3.2. Methodological Appraisal of Included Studies

A descriptive methodological appraisal of the ten included studies was undertaken using the Mixed Methods Appraisal Tool (MMAT), version 2018. Consistent with the exploratory purpose of this scoping review, the MMAT was used to identify common methodological strengths and limitations across the included quantitative and mixed-methods studies; it was not used as a formal design-specific risk-of-bias assessment to exclude studies or to weight the narrative synthesis.

**Figure 2:**
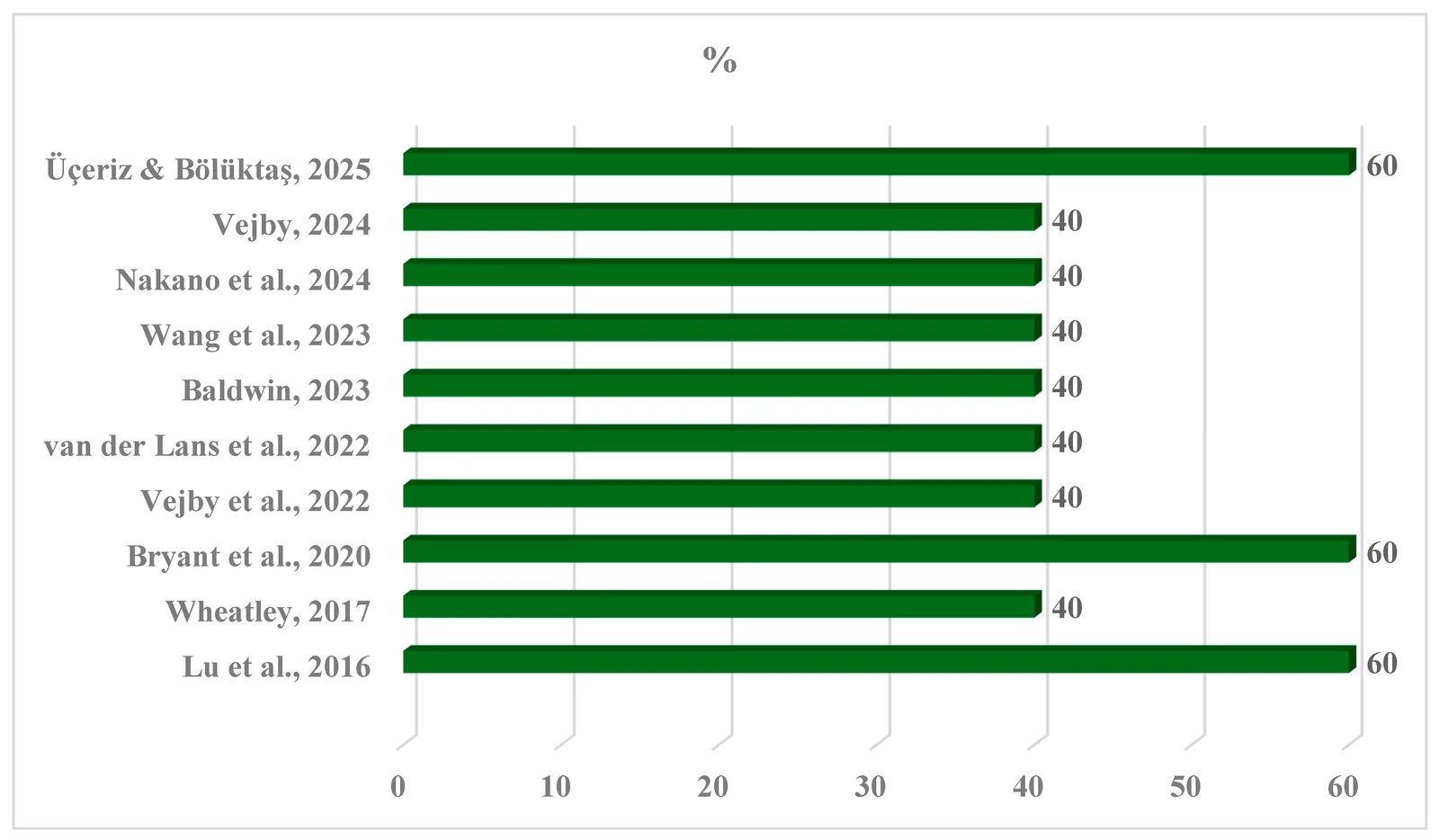
Summary of descriptive methodological appraisal of the included studies using the Mixed Methods Appraisal Tool (MMAT), version 2018 [25,26,27,28,29,30,31,32,33,34].

The appraisal identified recurring limitations in the available evidence base. The three pilot randomized controlled trials had limited reporting of blinding and completeness of outcome data [25,26,27]. The seven non-randomized studies commonly had limitations related to representativeness, potential confounding, comparator groups, attrition, and completeness of follow-up reporting. Some studies reported the use of validated outcome measures and described intervention delivery; however, variation in study design, sample size, intervention content, and outcome measurement limited cross-study comparison [28,29,30,31,32,33,34]. These observations were used to contextualize the mapped evidence and interpret findings cautiously rather than to determine intervention-favorable findings. Details of the MMAT appraisal are presented in Table 2 and Figure 2.

### 3.3. Nurse-Led Interventions Used in Hematopoietic Cell Transplantation Recipients

The characteristics and reported outcomes of nurse-led interventions are summarized in Table 4. Most nurse-led interventions focused on education, counselling, and motivational support, with the aim of supporting HCT recipients in areas such as health-promoting behaviors [32], understanding of the HCT process [33], self-care [28], physical activity, and nutrition [29,34]. There was only one intervention that addressed symptom monitoring, reporting and management and this was when the HCT recipient was still in the hospital [26]. This intervention did not include follow-up after discharge. On the other hand, one study used an intervention that included patient navigation, consultation, coaching in self-management skills, referral to dieticians, physiotherapists, or psychologists [30], and this was the most comprehensive of all the interventions.

One nurse-led intervention focused on psychological well-being and quality of life through a healing-touch protocol delivered by certified nurse practitioners. The intervention incorporated techniques including pain drain, chakra connection, magnetic clearing, and mind clearing. A separate relaxation arm in the same study was delivered by a graduate clinical psychology student and was therefore not considered a nurse-led intervention in this review [25], along with another study focusing on Mandala art therapy that integrated music and meditation [27]. Another intervention focused on improving aspects related to advance care planning for HCT recipients and this used an advance care planning (ACP) bundle to promote assessment, documentation and regular update of the patient’s ACP needs [31]. End-of-life situations are common in cancer and chronic disease management, and the limited number of interventions addressing advance care planning highlights an area for future nurse-led intervention development and evaluation.

It is evident from the above summary that aspects such as survivorship care, stigma, social isolation, fear of cancer recurrence, cancer screening, symptom management, palliative care, information needs, and others in the realm of nursing care have not been adequately targeted by nurse-led interventions. These gaps identify priority areas for future intervention development and evaluation, including survivorship care, social isolation, fear of cancer recurrence, symptom management, palliative care, and unmet information needs.

The nurse-led interventions also relied mostly on old tools such as pamphlets, telephone follow-up [29,33,34], diet diaries [33], and emails or websites [26]. Nurse-led interventions were delivered through face-to-face education and counselling, printed educational materials, telephone follow-up, diet diaries, email communication, and web-based resources. Moreover, HCT is usually performed in well-equipped inpatient hospital settings where the costs are usually very high, especially for populations in LMIC settings. No included study specifically evaluated nurse-led interventions targeting cost of care, outpatient HCT delivery, or medication adherence as outcomes. These areas may warrant future research, particularly where service capacity and resource constraints influence access to HCT. It is important to note that outpatient HCT is feasible and can have similar outcomes to inpatient HCT, when the patients are selected well following rigorous eligibility criteria.

### 3.4. Reported Favorable Findings of the Nurse-Led Interventions Used in HCT Recipients

The nurse-led interventions demonstrated both potential and limitations, as indicated by the summary presented in Table 5. Some nurse-led interventions were able to improve process-of-care-related outcomes. For instance, the ACP follow-up bundle was effective at improving documentation of HCT recipients’ ACP needs and decreasing nurses’ perceptions of barriers to ACP [31]. And the healing touch and relaxation protocol led to significant decreases in length of hospital stay [25], while the BMT class education program significantly reduced the number of 30-day readmissions after HCT [28]. The healing touch and relaxation protocol was unable to lead to significant changes in depression and QOL [25], while the BMT class education program did not have a significant impact on the length of stay after HCT and infection rate [28].

Other interventions demonstrated potential benefit in terms of improving symptoms of fatigue [26,33], vomiting [26], QOL [30], anxiety, and hopelessness [27,32], and self-care activities and self-care efficacy [26,29,30,32,34]. It should be noted that most of the studies that measured and aimed at improving depression, anxiety, and QOL did not demonstrate significant improvements on these outcomes [25,29,33]. The outcomes of the nurse-led interventions could have been affected by the lack of adequate follow-up periods, design issues, and the quality and efficacy of the interventions. To be more effective, nurse-led interventions for HCT recipients must be more comprehensive to address the impact of the prolonged and strenuous treatments, lengthy hospitalizations, numerous physical symptoms, self-care deficiencies, psychological distress, and low levels of positive psychological well-being.

## 4. Discussion

This scoping review found ten studies using nurse-led interventions for HCT recipients. The findings of the review show a map of the approaches and techniques nurses have so far utilized to deliver a range of supportive, educational, psychosocial, and care coordination interventions to address selected clinical and patient-reported outcomes. Across the ten studies, the overall methodological quality was moderate, and most were implemented at a single site using quasi-experimental designs or pilot randomized trials, which limits the strength of causal inference, but provides a foundation for developing more robust and efficacious nurse-led interventions and models of care in HCT.

The included interventions clustered around core transplant nursing functions such as patient preparation and education before HCT [28,33], symptom monitoring and management [25,26], health-promoting behaviors such as physical activity and nutrition [29,33], psychological and spiritual support [25,27], navigation and survivorship follow-up [30], and advance care planning [31]. This demonstrates heterogeneity in content and intensity, and the diversity of the nurse’s role in transplant care. Most interventions demonstrated feasibility and improved at least one targeted outcome (for example, self-care self-efficacy, physical activity, selected symptoms, or advance care planning documentation), but effects on the broader outcomes such as global quality of life, psychological distress, symptom burden, and longer-term recovery were generally limited or lacking. This was especially common when interventions were brief, lacked adequate follow-up, or limited to a single phase of the HCT trajectory [25,26,29,31,32]. Across the included studies, nurse-led interventions demonstrated preliminary feasibility and were associated with favorable findings in selected outcome domains, including self-care self-efficacy, physical activity, symptom burden, and advance care planning documentation. However, these findings should be interpreted cautiously. The studies varied substantially in intervention content, timing, delivery mode, outcomes assessed, and methodological design; most were small, single-center pilot, quality-improvement, or quasi-experimental studies. Consequently, the current evidence does not establish causal effects or allow conclusions about the comparative potential benefit of specific nurse-led intervention components.

This pattern is consistent with wider cancer survivorship evidence, where self-management interventions frequently improve self-efficacy and selected symptoms but show variable and sometimes limited effects on overall quality of life, due to limited intervention content and “active ingredients”, and lack of a multicomponent strategy [35,36,37]. Effective self-management programs are those that typically combine information, skills training, problem-solving and ongoing support rather than one-off education [38]. Thus, future HCT nurse-led interventions need to move beyond single-component designs to achieve more durable benefits.

Several studies highlighted the potential of nurse-led programs to improve symptom control and self-management. Lu et al. (2016) tested healing touch and relaxation therapy, which were highly acceptable and produced small benefits in outcomes such as length of hospital stay but not in mood or overall quality of life [25]. In contrast, the electronic symptom-monitoring intervention by Bryant et al. (2020) showed that nurse-driven review of patient-reported symptoms during hospitalization is feasible and improves symptom visibility and prompt management, although the use of a pilot design limited evidence about outcomes such as complications or readmissions [26]. It has been entrenched elsewhere that self-management interventions for cancer survivors using multicomponent programs can reduce pain and other symptoms and improve self-efficacy, but their effects on quality of life are inconsistent [37,39,40].

Lifestyle-oriented interventions in this review focused mainly on nurse-led physical activity and nutrition counselling during inpatient isolation, using individualized goals to promote mobilization and maintain intake [29,34]. These feasibility studies showed that a structured supportive intervention can be integrated into routine HCT nursing care and can improve levels of physical activity and nutrition status, but due to the small samples, the interventions’ effects on fatigue, functional status, and body composition remain unclear [29,34]. Similarly, health-promoting nursing intervention targeting education and support can enhance self-care self-efficacy and modify biological markers related to anxiety, suggesting a promising mechanistic pathway for nurse-led care on both subjective and objective patient outcomes [32]. These findings align with wider oncology work showing that clearly specified self-management support practices are crucial for translating interventions into routine care [41,42].

Psychological and existential concerns are central to the HCT experience, yet few included interventions were designed specifically for these domains. Lu et al. (2016) used healing touch and relaxation, and Üçeriz and Bölüktaş (2025) tested an art-based mandala intervention; both showed that nurse-delivered complementary therapies are acceptable and can improve selected psychological or spiritual outcomes, but sample sizes were small and long-term or subgroup effects were unclear [25,27]. This pattern is consistent with wider psychosocial oncology research, where single-modality interventions often have modest effects unless embedded in broader survivorship care models. Uncertainty about the transplant process and outcomes was explicitly targeted in one pre-transplant nursing intervention, which aimed to prepare patients more comprehensively for HCT [33]. The intervention demonstrated reduced uncertainty and improved preparedness, supporting the value of structured, nurse-led pre-transplant education in addressing cognitive and emotional information needs beyond standard information provision [33]. HCT research focusing on caregivers shows that structured psychological and mobile-health interventions can reduce burden, depression, and post-traumatic stress symptoms in family members, highlighting an important but largely untapped arena for nurse-led models [43,44], and utilization of emerging technologies in nurse-led interventions of HCT recipients.

Across the ten included studies, most interventions relied on traditional modalities such as in-person sessions, printed materials, telephone calls, and basic web resources [28,29,31,33,34]. There was minimal use of telehealth platforms, mobile health applications, artificial intelligence, or integrated digital decision support, despite growing evidence that nurse-led telehealth interventions can effectively support symptom management and self-care for patients receiving cancer therapies [45,46]. Recent nurse-led or nurse-supervised telehealth models, including post-acute transition programs and digital-plus-nurse support packages, have shown benefits in terms of symptom burden, patient activation, and reduced rehospitalization in broader oncology and palliative care populations [47,48]. Several interventions used telephone follow-up, email communication, and web-based resources to support care delivery. However, more advanced digital approaches, including mobile-health applications, telehealth platforms, and artificial intelligence-enabled tools, were not identified among the included nurse-led intervention studies. The absence of artificial intelligence-enabled interventions in the included literature may represent an opportunity for future research; however, such tools require evaluation for safety, equity, usability, and clinical appropriateness in HCT settings [47,48].

In HCT specifically, early work is also exploring innovative psychosocial technologies such as virtual-reality-based supportive interventions for transplant recipients and app-based psychosocial support for caregivers, underlining how rapidly the technological landscape is evolving [49]. Against this backdrop, the almost complete absence of technology-enabled nurse-led models in the current set of HCT interventions indicates a major opportunity to extend nursing support across settings and time, particularly during transitions from hospital to home and through the prolonged survivorship phase, and in LMIC settings.

### 4.1. Gaps and Implications for Future Nurse-Led Models

Across all ten studies, several consistent gaps emerged. First, most interventions were conducted in high-income countries and no nurse-led trials reported from LMIC settings. Second, the interventions tended to be time-limited and tethered to a single phase (pre-transplant, inpatient, or early follow-up), rather than being designed as longitudinal programs that span the entire transplant trajectory [25,30,33]. Third, while several studies used telephone calls or basic electronic tools, none fully leveraged contemporary telehealth, mobile health, or advanced digital platforms to extend nurse-led support beyond the hospital or to enable continuous monitoring, tailored coaching, or follow-up care and support [26,34].

Taken together, the evidence synthesized in this scoping review suggests that nurse-led interventions in HCT are both feasible and acceptable and can improve specific outcomes related to symptoms, self-management, physical activity, and care processes, but current models remain fragmented and under-powered to transform long-term recovery and quality of life. Building on insights from broader oncology, future research should prioritize multicomponent, phase-based nurse-led programs that address symptom management, self-management, psychological and existential needs, caregiver support, and advance care planning across the HCT trajectory. Hybrid in-person and digital delivery models, including telephone, telehealth, and mobile-health approaches, should be evaluated to improve continuity and access to care. Artificial intelligence-enabled tools may also warrant investigation, but their safety, equity, usability, clinical appropriateness, and performance across diverse HCT populations should be established before implementation. Future studies should use adequately powered, multicenter designs and assess effectiveness, feasibility, implementation fidelity, and sustainability across diverse health-care settings. Rather than confirming potential benefit, this review maps promising areas for intervention development and identifies priorities for larger, multicenter, controlled, and longitudinal evaluations.

The findings of this scoping review should be interpreted as a map of the current evidence rather than confirmation of intervention reported favorable findings. Although several studies reported favorable changes in selected outcomes, the evidence base was small and heterogeneous, and most studies used single-center, pilot, quality-improvement, or quasi-experimental designs with modest sample sizes. Differences in intervention content, timing, delivery mode, comparators, and outcome measures precluded pooled analysis and limit causal inference. Therefore, the results identify promising intervention domains for future development and evaluation rather than specific models ready for routine implementation.

### 4.2. Relevance to Clinical Practice

The findings should not be interpreted as supporting routine implementation of any specific nurse-led intervention. Rather, the mapped evidence identifies candidate components for future intervention development, including structured pre-transplant education, self-management support, symptom monitoring, physical activity and nutrition support, psychosocial care, care navigation, survivorship follow-up, and advance care planning. Future interventions should be co-designed with HCT recipients, family caregivers, transplant nurses, and multidisciplinary teams; adapted to local service capacity and patient needs; and evaluated initially for feasibility, acceptability, fidelity, equity, and resource implications. Promising interventions should subsequently be tested in adequately powered, multicenter, controlled, and longitudinal studies using a core set of patient-reported, clinical, and health-service outcomes before broad implementation is considered.

### 4.3. Strengths and Limitations of the Study

This scoping review has several strengths. To our current knowledge, this is the first review to systematically map nurse-led interventions specifically for adult HCT recipients across the full care continuum, using a rigorous, pre-registered protocol and contemporary scoping review guidance for searching, selection, charting, and narrative synthesis. The use of multiple databases and grey-literature sources, duplicate screening and data charting by independent reviewers, and structured quality appraisal with the MMAT enhanced the transparency, reproducibility, and credibility of the findings. A further strength is the detailed description of intervention characteristics, contexts, and outcomes, which makes the review directly useful for clinicians and researchers seeking to design or adapt nurse-led models of care for HCT recipients.

The evidence base mapped in this scoping review has important limitations. Only ten studies were included, most of which were small, single-center evaluations from high-income countries using pilot or quasi-experimental designs. This limited and heterogeneous evidence base restricts generalizability across transplant programs and health systems and precludes meta-analysis. The English-language, full-text restriction may have introduced language bias and excluded relevant interventions reported in other languages or formats. In addition, limiting the search to 2015–2025 focused the review on contemporary HCT practice but may have omitted earlier foundational work. Publication bias and selective outcome reporting are also possible, as feasible or favorable short-term interventions may be more likely to be published, while longer-term clinical outcomes were inconsistently reported. Variation in intervention content, timing, delivery, and outcomes further limited direct comparison. As a scoping review, this study maps evidence rather than establishes effectiveness; findings should therefore be interpreted cautiously as hypothesis-generating. Finally, the review was initially registered in PROSPERO as a systematic review and was refined to a scoping review after substantial heterogeneity became apparent; although descriptive, non-pooled synthesis was planned from the outset, this refinement should be considered when interpreting the final report.

## 5. Conclusions

This scoping review mapped a small and heterogeneous body of evidence on nurse-led interventions for adult HCT recipients. The included studies provide promising preliminary findings that nurse-led education, self-management support, symptom-focused care, navigation, psychosocial interventions, and advance care planning may support selected patient and care-process outcomes. However, the evidence is insufficient to establish potential benefit because of the limited number of studies, small samples, single-site settings, and predominance of pilot and quasi-experimental designs. Future research should use robust, adequately powered, multicenter, and longitudinal designs to evaluate intervention feasibility, implementation, equity, and patient, clinical, and health-service outcomes across the HCT trajectory, including in resource-limited settings.

## Figures and Tables

**Table 1:** Search strategy (summary).

Keywords:	*Transplant population: bone marrow transplant, hematopoietic stem cell transplant, HCT, hematopoietic cell transplant, stem cell transplant, autologous transplant, allogeneic transplant.* *Nurse-led interventions: nurse-led, nursing intervention, nurse-managed, nurse-delivered, clinical nurse specialist, transplant nurse, oncology nurse, advanced practice nurse, nurse navigator.* *Outcomes: symptom, symptom burden, pain, fatigue, psychological distress, anxiety, depression, self-care, self-management, quality of life, health-related quality of life, patient-reported outcomes, treatment-related complications, infections, graft-versus-host disease, functional status, treatment adherence, medication adherence, survival, readmission, healthcare utilization.*
Generic search string used across databases:	(bone marrow OR “hematopoietic stem cell” OR HCT OR “stem cell” OR “hematopoietic cell”) N3 (transplant * OR transplantation) AND (nurse-led OR “nurse led” OR “nursing intervention *” OR “nurse-managed” OR “nurse managed” OR “nurse-delivered” OR “nurse delivered” OR “transplant nurse *” OR “oncology nurse *” OR “clinical nurse specialist *” OR “advanced practice nurse *”) AND (symptom * OR pain OR fatigue OR “psychological distress” OR anxiety OR depression OR “self-care” OR “self-care” OR “self-management” OR “self-management” OR “quality of life” OR HRQoL OR “patient-reported outcome *” OR PRO * OR “treatment-related complication *” OR infection * OR “graft versus host” OR GVHD OR survival OR “treatment adherence” OR “medication adherence” OR “healthcare utilization” OR “healthcare utilization”).
Corresponding subject-heading terms, including descriptors	*Hematopoietic Stem Cell Transplantation; Bone Marrow Transplantation; Nursing; Oncology Nursing; Hematologic Neoplasms; Signs and Symptoms; Pain; Fatigue; Self Care; Patient Education; Quality of Life; Patient Reported Outcome Measures; Treatment Adherence and Compliance; Medication Adherence; Health Services Utilization, adapted for each database’s thesaurus where available. No language restrictions were applied during searching; for feasibility, screening was restricted to full-text articles in English, a common pragmatic approach in scoping reviews. The main keyword and subject-heading blocks are summarized in the manuscript, and full database-specific strategies are provided in a Supplemental File to support reproducibility.*

* Truncation (e.g., intervention *, transplant *, PRO *).

**Table 2:** Design-specific descriptive methodological appraisal of included studies using the Mixed Methods Appraisal Tool (MMAT), version 2018.

Study	Study Design	MMAT Category Applied	Appraisal Summary
Lu et al. [25], 2016	Randomized two-group prospective study	Randomized controlled trial	A two-group randomized design was reported. The study was appraised with respect to the appropriateness of randomization, comparability of groups at baseline, completeness of outcome data, blinding of outcome assessors, and adherence to the assigned intervention. As a small prospective study, its findings should be interpreted with consideration of the reporting of allocation procedures, assessor blinding, outcome-data completeness, and intervention adherence.
Bryant et al. [26], 2020	Pilot two-arm randomized controlled trial	Randomized controlled trial	A pilot two-arm randomized design was reported. Appraisal addressed randomization procedures, baseline comparability, completeness of outcome data, outcome-assessor blinding, and adherence to the assigned intervention. The pilot nature and likely small sample limit statistical precision and generalizability; conclusions should therefore be interpreted as preliminary.
Üçeriz & Bölüktaş [27], 2025	Parallel-group pilot randomized controlled trial	Randomized controlled trial	A parallel-group pilot randomized design was reported. Appraisal addressed randomization, baseline comparability, completeness of outcome data, outcome-assessor blinding, and intervention adherence. The study provides preliminary comparative evidence, although the pilot sample size may limit precision and the certainty of estimates.
Wheatley [28], 2017	Retrospective quality-improvement project	Non-randomized study	The retrospective quality-improvement design evaluated outcomes in a routine clinical setting. Appraisal addressed participant representativeness, appropriateness of intervention and outcome measurements, completeness of outcome data, confounding, and whether the intervention was administered as intended. The retrospective pre–post approach, without random allocation, may be affected by selection bias, secular trends, and unmeasured confounding.
Vejby et al. [29], 2022	Non-equivalent-group pre-test–post-test study	Non-randomized study	The study used a non-equivalent comparison-group pre-test–post-test design. Appraisal addressed participant representativeness, appropriateness of intervention and outcome measurements, completeness of outcome data, confounding, and intervention delivery. Because group allocation was non-random, baseline differences and residual confounding may have influenced the observed outcomes.
van der Lans et al. [30], 2022	Mixed-methods study	Mixed methods	The single-group pre-test–post-test design assessed outcomes before and after the intervention. Appraisal addressed participant representativeness, measurement appropriateness, completeness of outcome data, confounding, and intervention delivery. Without a comparator group, temporal effects, regression to the mean, and unmeasured confounding may have contributed to observed changes.
Baldwin [31], 2023	Quality-improvement pre–post study	Non-randomized study	Quantitative and qualitative components were used to investigate complementary aspects of the intervention. Appraisal considered the quality of each component and the rationale for mixed methods, integration and interpretation of findings, and handling of divergences between quantitative and qualitative results. The contribution of the mixed-methods design depends on transparent integration of the component findings.
Wang et al. [32], 2023	Quasi-experimental pre-test–post-test study	Non-randomized study	This quality-improvement pre–post study examined outcomes following implementation in a clinical setting. Appraisal addressed participant representativeness, measurement appropriateness, completeness of outcome data, confounding, and intervention delivery. In the absence of a concurrent comparator, temporal changes and other uncontrolled confounders may have influenced the observed findings.
Nakano et al. [33], 2024	Single-group pre-test–post-test study	Non-randomized study	The quasi-experimental pre-test–post-test study evaluated change after implementation of the intervention. Appraisal addressed participant representativeness, measurement appropriateness, completeness of outcome data, confounding, and intervention delivery. The absence of random allocation and potential uncontrolled confounding limit causal interpretation of pre–post changes.
Vejby [34], 2024	Feasibility intervention study with interviews	Mixed methods	Quantitative feasibility outcomes and qualitative interview data were used to examine intervention feasibility and participant experience. Appraisal considered the quality of the quantitative and qualitative components, the rationale for using mixed methods, integration and interpretation of findings, and treatment of inconsistencies between components. The lack of a comparator group limits conclusions about intervention effectiveness.

**Table 3:** Characteristics of the studies included in the review.

Author, Year	Country	Design	Setting	Sample	Mean Age
Lu et al. [25], 2016	USA	A randomized, two-group prospective design	Blood and Bone Marrow Transplant Unit of a University Hospital	Healing-touch group (n = 13)	57.62 ± 7.67
				Relaxation completers (n = 13)	55.77 ± 5.56
				CG (n = 20	57.25 ± 7.25
Wheatley [28], 2017	USA	A quality-improvement project using a retrospective analysis	Cancer Center	IG (n = 33)	55.1 ± 11.3
				CG (n = 35)	56.8 ± 12.2
Bryant et al. [26], 2020	USA	A prospective, single-site (1:1) pilot randomized two-arm study	Cancer Center	IG (n = 38)	51.3 ± 13.6
				CG (n = 38)	51.1 ± 13.7
Vejby et al. [29], 2022	Sweden	Quasi-experimental non-equivalent group (pre/post-test) design	Hematology Department of a Hospital	IG (n = 21)	52 ± 14.7
				CG (n = 22)	52 ± 15.7
van der Lans et al. [30], 2022	The Netherlands	Mixed methods	Outpatient	IG (n = 199)	Median = 56
				CG (n = 62)	Median = 62
Baldwin [31], 2023	USA	Quasi-experimental quality-improvement project using a pre–post design	Comprehensive Cancer Center	Nurses (n = 10)	Not reported
				Patients (n = 14)	
Wang et al. [32], 2023	China	Quasi-experimental (pre/post-test) design study	Bone Marrow Transplant Center	IG (n = 30)	41.20 ± 12.86
				CG (n = 30)	44.46 ± 9.75
Nakano et al. [33], 2024	Japan	Pre/post-test single group design	University Hospital	IG (n = 30)	Median = 52
Vejby [34], 2024	Sweden	Single-arm intervention and interview study (mixed methods)	Hematology Department of a Hospital	IG (n = 20)	56
Üçeriz & Bölüktaş [27], 2025	Türkiye	Parallel-group pilot randomized controlled trial	University Hospital’s Bone Marrow Transplant Unit	IG (n = 23)	Not reported
				CG (n = 23)	

IG, intervention group; CG, control group.

**Table 4:** Nurse-led interventions and their outcomes.

Author (Year)	Nurse-Led Intervention and Comparator	Outcomes/Instruments		Key Findings	Limitations
Lu et al. [25], 2016	Healing-touch intervention: Daily 20 min healing-touch sessions beginning one day after stem cell transplantation, delivered by two certified nurse practitioners, in addition to usual care. Techniques included pain drain, chakra connection, magnetic clearing, and mind clearing. Comparator: Usual care, including nutritional and hydration support, pain management, protective isolation, physical rehabilitation, recreational therapy, mental-health support, and pastoral care. CG: Usual care includes support services related to nutritional and hydration support, pain management, protective isolation, physical rehabilitation therapy, recreational therapies, mental health, and pastoral support.	Length of hospital stay; time to engraftment; mood measured using the Profile of Mood States–Short Form; depression measured using the Center for Epidemiologic Studies Depression Scale; quality of life measured using the functional assessment of cancer therapy–bone marrow		Participants receiving healing touch were discharged, on average, two days earlier than those receiving usual care; however, no statistically significant differences were reported for mood, depression, quality of life, or 100-day readmissions.	Single-site study; small sample; lack of randomization; intervention required daily 20 min sessions
Wheatley [28], 2017	IG: The Bone Marrow Transplant (BMT) Basics Class is a nurse-led educational program dedicated to preparing patients and their caregivers for the treatment outline and self-care behaviors associated with receiving a BMT. CG: Usual care.	Infection rate Length of stay (LOS) 30-day readmission rate Patient satisfaction information	Hospital Consumer Assessment of Healthcare Providers and Systems (HCAHPS)	No significant statistical differences between the pre- and post-implementation groups in LOS. The 30-day readmission rate was lower among the post-implementation group (7.69% vs. 2.56%), with fewer patients readmitted for infection. There was an increase in CLABSI rate in the post-implementation group.	No randomization Small sample Unclear control group
Bryant et al. [26], 2020	IG: Patients reported daily on 16 common symptoms using the PRO version of the Common Terminology for Adverse Events (PRO-CTCAE) at admission, and daily until discharge. Patients also reported symptoms to the nurse daily. A report about the symptoms was generated on the study web page. Each morning, an email containing a link to the page was sent to the nurse for the patient. Reports showing the most severe symptoms on top were printed for use in morning rounds and to guide conversations with patients and direct the plan of care. CG: Patients completed the PRO-CTCAE at baseline, and on day 7, 10, and 14 during their hospitalization. Patients also reported symptoms to the nurse throughout the day.	Symptomatic adverse events Comorbidity index	PRO-CTCAE survey Hematopoietic cell transplantation comorbidity index (HCT-CI)	After day seven, 92% of IG reported fatigue compared to 100% in the CG (*p* < 0.05). On day 10 no one in the IG reported vomiting compared to 36% in CG (*p* = 0.002). IG experienced lower peak symptom burden (average of 16 symptoms) on day 7, 10, and 14 compared to CG (*p* = 0.03). After controlling confounders (age, transplant type, conditioning type, HCT-CI, and symptom burden at baseline), CG had a higher mean peak symptom burden compared to IG (*p* = 0.08).	Small sample Single site Electronic symptom reporting may inflate results
Vejby et al. [29], 2022	IG: Delivered by a nurse and composed of pre-transplantation consultation, daily support in the hospital, and post-discharge consultation. The intervention was based on motivational interviewing (MI) and encouraged being physically active before the transplant. Daily support comprised standardized goals such as sitting in a chair, resistance band exercise, and others. Goals were followed every day by the nurse, who gave advice and encouragement using MI. Phone call follow-up by nurse at day 14 post-discharge to assess physical activity and encourage an active lifestyle. CG: Patients were informed that physical activity is important for people undergoing hematopoietic stem cell transplantation and instructed to keep an activity diary during their entire hospital stay (no extra physical activity support was provided).	Exertion during activities Energy used by the body during physical activity Physical capacity Health-related QOL	The Borg Rating of Perceived Exertion (RPE) Metabolic equivalent (METs) of tasks Six-minute walk test (6MWT) Functional assessment of cancer therapy-anemia (FACT-An)	IG was more physically active than CG (*p* = 0.05). IG was less bedridden per day compared to CG (*p* = 0.03). No differences in the number of activities out of bed or perceived exertion on the Borg RPE scale. Mean daily METs were higher in the IG compared to CG (*p* = 0.02). Mean daily METs from sitting in a chair were higher in IG compared to CG (*p* = 0.05). No differences regarding METs from light to moderate intensity activity. IG walked longer (555 m) than CG (488 m) *p* = 0.03, but there was no difference at discharge (*p* = 0.42). No differences in health-related QOL between groups.	Self-reported measures of physical activity Single site Small sample Inequivalent samples at baseline
van der Lans et al. [30], 2022	IG: Received standard care plus patient navigation provided by three bachelor-level cancer nurses specialized in hemato-oncology. Provided consultation in the first year after HCT. Patient navigation consisted of two 45 min face-to-face consultations with a cancer nurse in the first year of HCT (3 to 5 months and one year). Before consultations, psychological distress was assessed, and other problems. During consultation, patients were encouraged to prioritize the problems and define goals, and were coached in self-management skills to improve QOL and post-transplant health. When needed, patients were referred to dieticians, physiotherapists, or psychologists. CG: Received standard care by hematologist.	QOL Effect of cancer therapy on physical, social/family, emotional, and functional well-being Self-management Quality of patient-centered care Self-management knowledge and behavior Self-efficacy for managing chronic disease	EORTC QLQ-30 Functional assessment of cancer therapy bone marrow transplant (FACT-BMT) Domain of daily effect on daily life (investigator-developed tool) Consumer assessment of health plan survey (CAHPS) Partner in Health Scale (PIH) Self-efficacy for managing chronic disease six-item scale	In the first year, in the IG, the QOL domains of physical, role, and social functioning and symptom scores all improved significantly (*p* < 0.01). In the first year, in the IG, FACT-G domains related to physical, social, and functional well-being, along with additional concerns, BMT, and FACT-BMT, all significantly improved (*p* < 0.01). No significant difference in the FACT-DMT domain between groups. IG had significantly higher total self-efficacy (*p* = 0.026). No significant differences between groups in self-management knowledge and behavior and patient-centered care. IG experienced improvements in daily activities, social contacts, sexuality, leisure activities, practical matters in daily life (*p* < 0.01), transport and mobility (*p* < 0.030), finances (*p* < 0.002), dealing with symptoms, side effects, and emotions, and spirituality (*p* < 0.001).	No randomization
Baldwin et al. [31], 2023	IG: The advance care planning (ACP) follow-up bundle was developed from the literature, including national guidelines and unit-based needs, and informed by the Advance Care Planning Framework published by the National Health Service. The bundle includes five elements: (1) unit-based education, (2) nurse reminder system to alert nurses to new allogeneic admissions, (3) assessing for patient-reported changes from pre-admission ACP session during admission, (4) documenting ACP needs using the ACP template note, and (5) charting provider notification as needed using the electronic medical records system.	ACP follow-up documentation Nurse confidence and frequency of ACP use, and assess barriers Frequency of engaging in ACP; barriers to utilizing ACP	Electronic medical record of the patient Confidence in engaging in ACP (six Likert scale questions assessed confidence in discussing, documenting, advocating, and educating on ACP) Two Likert scale questions assessed the frequency of nurse-driven ACP discussions and documentation of ACP needs (Scores range from 1 = very confident to 5 = unconfident)	ACP documentation increased from 0 at baseline to 100% in 6 weeks. Nurses’ confidence about ACP increased significantly (*p* < 0.01). Frequency of ACP discussions increased significantly (*p* = 0.024), resulting in an overall significant increase in discussing ACP over the intervention period (*p* = 0.022). Documentation of patient ACP needs increased significantly (*p* < 0.001). At baseline, the most common barriers were lack of time (100%), ACP training (100%), familiarity with finding ACP documents (70%), provider encouragement or response (50%), and clear guidelines on which patients should receive ACP (30%). Post-intervention, the number of nurses who reported a lack of ACP training(30%), time (50%), and familiarity with finding ACP documents (20%) declined. The number of nurses reporting a lack of provider encouragement increased to 70%.	IG: The advance care planning (ACP) follow-up bundle was developed from the literature, including national guidelines, unit-based needs, and informed by the Advance Care Planning Framework published by the National Health Service. The bundle includes five elements: (1) unit-based education, (2) nurse reminder system to alert nurses to new allogeneic admissions, (3) assessing for patient-reported changes from pre-admission ACP session during admission, (4) documenting ACP needs using the ACP template note, and (5) charting provider notification as needed to use the electronic medical records system
Wang et al. [32], 2023	IG: Received training in health promotion strategies. CG: Usual care.	Self-efficacy of self-care Testing for genes related to anxiety	Strategies used by people to promote health tools (SUPPH) Blood samples for genetic testing	Total self-efficacy and its domains of compatibility, decision making, stress reduction, and life enjoyment were significantly higher in the IG compared to the CG (*p* < 0.01). The expression of the gene directly related to anxiety, 5-hydroxytryptamine receptor (5-HT1A) and corticotropin-releasing hormone receptor 1 (CRHR1) decreased significantly in the IG after the intervention (*p* = 0.01).	Small sample Single site Poorly described intervention
Nakano et al. [33], 2024	Intervention included: Provided uncertainty assessment, then information using a pamphlet and nurse-led explanations to reduce pre-transplant uncertainty and anxiety. Discussing individual patient schedules from admission until discharge. Verifying patient understanding of information and providing emotional support. Patients received positive feedback and at least one telephone call to follow up on uncertainty and to provide support.	Uncertainty in illness QOL Anxiety Depression	Uncertainty in illness scale (UUIS) European Organization for Research and Treatment of Cancer QOL Questionnaire (EORTC QLQ-C30) Hospital anxiety and depression scale (HADS)	Overall uncertainty decreased significantly after the intervention (*p* < 0.001). All the six subscales of the UUIS—unpredictability of daily life (*p* < 0.001), ambiguity of characteristics of illness (*p* = 0.001), complexity of interpretation of information (*p* = 0.002), lack of cues to find meaning in illness (*p* = 0.005), instability of self-confidence in carrying on a struggle against illness (*p* = 0.011), and unpredictability of recovery from illness—significantly declined post-intervention. There was no significant change in QOL domains of physical functioning, role functioning, emotional functioning, cognitive functioning, social functioning, and global health status. On the symptom scale, there was a significant change in fatigue (*p* < 0.05) and constipation (*p* = 0.035). There was a significant decrease in overall psychological distress (*p* = 0.029), but no change in the level of anxiety and depression.	Singel site Small sample No randomization Short follow-up period
Vejby [34], 2024	IG: The intervention comprised the same physical activity support and supported nutritional intake in using a self-reported diet diary, dietician consults, and nutritional follow-up by the study nurse. Nutritional counselling by a dietician occurred twice and was based on a 3-day food diary. At the pre-transplant visit, the counselling was characterized by the offering of individual advice to optimize nutritional status before HCT through a diet rich in calories, protein, and fluids. Eight days after stem cell infusion, advice was given to minimize weight loss and maintain energy and protein intake, with the dietician offering suggestions regarding tolerable supplements better than usual food. Daily follow-up on nutrition status by study nurse using standard care procedures and the dietician’s advice. The same day participants started experiencing eating problems, the study nurse provided additional support by recommending more energy- or protein-dense foods, nutritional drinks, and supplements for participants to taste. The study nurse also initiated or took part in clinical decisions regarding remedies for nutritional problems and parenteral nutrition, both with the participants and the physician. Following discharge, the study nurse followed up on the nutritional intake and physical activity prescription by phone at 14 days and 6 months post-transplant.	Self-reported physical activity Diet diary Physical activity goals setting; e.g., sitting goal, walking goals, outdoor activity goals, and others Six-minute walk test (6MWT)	Diet diary 6MWT Semi-structured interview	Adherence to the diet during hospitalization was not possible. All participants set physical activity goals, received daily (weekday) follow-up visits from the study nurse and kept an activity diary during hospitalization. The goals were generally not time-bound and comprised sitting time and daily walks. A majority spent an average of 42 min being physically active at a moderate to vigorous intensity, expressed as 12–18 on the Borgs RPE scale. All participants reported daily walks and were physically active for 1 h a day. Participants increased walking distances by an average of 37 m from pre-transplant to admission. Nutritional intake: participants were monitored twice a week from admission to discharge, and daily when they were not achieving their calculated intake goals two times in a row. Eight participants needed parenteral nutrition for 5–24 days.	No control group Small sample Sample from one site
Üçeriz & Bölüktaş [27], 2025	IG: Nurse-led art-based mandala intervention. Mandala is an occupation that relaxes the human mind in the form of meditation therapy that allows the individual to evaluate physical and psychological conditions, improve negative mood, and protect and improve psychological well-being. Participants were provided with ready-made stencil mandala coloring papers and 12 colored dry paints, crayons, and felt-tip crayons. The participants applied a mandala with ready-made stencil mandala coloring papers for a total of seven sessions for 30 min, accompanied by music containing nature or instrumental sounds. Music was used only during painting so that the patients did not feel as though they were in a hospital environment. CG: Standard care.	Psychosocial distress Psychological well-being Anxiety hope/hopelessness	Distress Thermometer Psychological well-being scale State-Trait Anxiety Inventory (STAI) Beck hopelessness scale	No difference in mean distress scores between groups. Hopelessness was lower in the IG than in the CG (*p* < 0.01). IG had increased psychological well-being on the day of transplantation, and after transplantation (*p* < 0.001). IG had a greater decrease in anxiety than the CG on the day of transplantation.	Singel site Small sample Unclear implementation of the intervention.

IG, intervention group; CG, control group; QOL, quality of life; HCT, hematopoietic stem cell transplantation.

**Table 5:** Potential benefits of nurse-led interventions.

Key Aspects of the Nurse-Led Intervention (Author)	Process-of-Care-Related Outcomes	Symptoms, QOL, Self-Care Practices, and Others
Healing-touch protocol [25]	(+) LOS after HCT	(−) Depression and QOL
Relaxation therapy protocol [25]	(+) LOS after HCT	(−) Depression and QOL
Bone marrow transplant class education program [28]	(+) Number of 30-day readmissions (−) LOS after HCT and infection rate	
Daily common symptom monitoring using a web page, email reports, and discussion during rounds and planning of care [26]		(+) Fatigue and vomiting (+) Overall symptom burden
Pre-HCT consultation, daily support while in the hospital using motivational interviewing, and post-discharge consultation [29]		(+) Physical activity and energy used during physical activity (−) QOL
Consultation, patient navigation, assessment of psychological distress, coaching on self-management, and referral to a dietician, physiotherapist, or psychologist as needed [30]		(+) QOL (physical, role, and social functioning) (+) Symptom burden and self-efficacy (+) Daily and leisure activity, sexuality, social contacts, mobility, dealing with symptoms, side effects, and emotions (−) Self-management knowledge and behavior
ACP follow-up bundle comprised unit-based education, nurse reminder systems, assessing change in patient ACP needs, documenting ACP needs, and chart provider notification [31]	(+) Documentation of ACP needs, and a decrease in perceived barriers to ACP among nurses	
Training patients in health-promoting strategies [32]		(+) Reduction in anxiety gene expression (+) Self-efficacy in decision making, stress reduction, and life enjoyment
Assessment of uncertainty and provision of information, emotional support, feedback, and telephone follow-up [33]		(+) Fatigue and uncertainty about illness (−) Anxiety, depression, and QOL
Physical activity and nutrition support, patient diet dairying, dietician consults, nutritional counselling, and follow-up [34]		(+) Moderate and vigorous physical activity (+) Need for parenteral nutrition
Art-based mandala that includes meditation, art-therapy, and music [27]		(+) Hopelessness, psychological well-being, and anxiety

(−), no improvement; (+) improvement; LOS, length of stay; QOL, quality of life; ACP, advance care planning; HCT, hematopoietic cell transplantation recipient.

## Data Availability

Data supporting the findings of this study are available within the article and its Supplementary Materials. The data charted from the included published studies are available from the corresponding author upon reasonable request.

## Supplementary Materials

The following supporting information can be downloaded at: https://www.mdpi.com/article/10.3390/nursrep16090344/s1, File S1: PRISMA-ScR Checklist; File S2: Detailed Database Search Strategy.

## Public Involvement Statement

There was no public involvement in any aspect of this research.

## Guidelines and Standards Statement

This scoping review was prepared following PRISMA guidelines, as well as the five-stage scoping review framework.

## List of Abbreviations

ACP	Advance Care Planning
BMT	Bone Marrow Transplantation
EBMT	European Society for Blood and Marrow Transplantation
GvHD	Graft-versus-Host Disease
HCT	Hematopoietic Cell Transplantation
HRQoL	Health-Related Quality of Life
IG	Intervention Group
JBI	Joanna Briggs Institute
LMICs	Low- and Middle-Income Countries
LOS	Length of Stay
MMAT	Mixed Methods Appraisal Tool
OM	Oral Mucositis
PCC	Population–Concept–Context
PRISMA	Preferred Reporting Items for Systematic Reviews and Meta-Analyses
PRISMA-ScR	PRISMA Extension for Scoping Reviews
PRO	Patient-Reported Outcome
QOL	Quality of Life
RCT	Randomized Controlled Trial
SWiM	Synthesis Without Meta-analysis
