# Supplementary material for: Nurse-Led Interventions and Outcomes in Adult Hematopoietic Stem Cell Transplant Recipients: A Scoping Review
Source: Nurs Rep. 2026 Sep 20;16(9):344. doi: 10.3390/nursrep16090344 (PMC13610441; doi:10.3390/nursrep16090344)
Supplement: Supplementary file 1 [file nursrep-16-00344-s001.zip › Detailed Database Search Strategy - Supplementary File S2.pdf]

Supplementary File S2 - Detailed Database Search Strategy

Title: Detailed Database Search Strategy - Nurse-Led Interventions and Health Outcomes Among Patients Undergoing Hematopoietic Stem Cell/Bone Marrow Transplantation

Review type: Scoping Review

Date range covered: 2015-01-01 to 2025-12-31

Prepared by: [Muliira JK & Lazarus ER], Senior Researchers

Search dates: EMBASE (2026-01-05); ProQuest (2026-01-05); CINAHL (2026-01-05); ScienceDirect (2026-01-05); Scopus (2026-01-05); Web of Science (2026-01-05); OpenGrey (2026-01-05)

Information sources: Searches were conducted via the Sultan Qaboos University Library discovery platform (e.g., EBSCO Discovery) and individual databases (EMBASE, CINAHL, Scopus, Web of Science, ScienceDirect, ProQuest) from 2015–2025.

Screening platform: Consensus (Consensus.app). References were exported in RIS/BibTeX, deduplicated in EndNote 21, and screened in Consensus by two independent reviewers.

Deduplication: EndNote 21 & manual verification

1. Scope and objectives

This supplemental file documents the complete database and grey literature search strategies used to identify primary empirical studies of nurse-led interventions aimed at improving health outcomes among patients undergoing hematopoietic stem cell or bone marrow transplantation. Searches spanned major bibliographic databases and grey sources. Publication dates were limited to 1 January 2015 through 31 December 2025 to map contemporary nurse-led interventions within current hematopoietic cell transplantation practice. This period was selected because it captures recent developments in transplant care and supportive-care delivery, including changing transplant approaches, expansion of outpatient and survivorship care, and increasing attention to self-management and technology-enabled care.

Population: Adult or pediatric patients undergoing hematopoietic stem cell/bone marrow transplantation (autologous or allogeneic)

Interventions: Nurse-led, nurse-coordinated, or nurse-delivered interventions (initiated, delivered, or primarily coordinated by nurses) across the transplant trajectory (pre-transplant, inpatient, and post-transplant follow-up)

Outcomes: Health outcomes including symptom burden, treatment-related complications, psychological outcomes, functional status, health-related quality of life, survival, and unplanned healthcare use (e.g., readmissions, emergency visits)

Study designs: Primary empirical studies (quantitative, qualitative, and mixed-methods)

2. Databases and platforms

- EMBASE (Embase.com or Ovid)
- ProQuest (Dissertations & Theses Global; Nursing & Allied Health collections)
- CINAHL (EBSCOhost)
- ScienceDirect (Elsevier)
- Scopus (Elsevier)
- Web of Science Core Collection (Clarivate)
- OpenGrey (grey literature repository)

Limits applied across databases where available:

- Publication dates: 2015-01-01 to 2025-12-31
- Humans
- Document types: Articles (and theses/reports for ProQuest/OpenGrey); reviews, protocols, editorials excluded at screening

Language: No limits at search; screening restricted to English for feasibility

3. Full search strategies (copy-paste ready)

A. EMBASE (Embase.com or Ovid)

Free-text:
“nurse-led” OR “nurse led” OR “nurse-managed” OR “nurse managed” OR “nursing intervention*” OR “nurse-driven” OR “nurse delivered” OR “clinical nurse specialist*” OR “advanced practice nurse*” OR APN* OR “registered nurse*” OR RN
AND
“bone marrow transplant*” OR “hematopoietic stem cell transplant*” OR “hematopoietic stem cell transplant*” OR HSCT OR “stem cell transplant*” OR “hematopoietic cell transplant*” OR “peripheral blood stem cell transplant*”
AND
“symptom*” OR “symptom burden” OR fatigue OR pain OR “graft versus host” OR “GVHD” OR “treatment-related complication*” OR infection*
AND
“quality of life” OR “health-related quality of life” OR HRQoL OR “functional status” OR “psychological distress” OR anxiety OR depression OR survival OR “overall survival” OR “event-free survival” OR “readmission*”

Subject headings (Emtree):
‘bone marrow transplantation’/exp OR ‘hematopoietic stem cell transplantation’/exp
AND ‘nursing’/exp

AND (‘quality of life’/exp OR ‘survival’/exp OR ‘treatment outcome’/exp OR ‘symptom assessment’/exp OR ‘mental health’/exp)

Limits: Publication year: [2015-01-01]/py TO [2025-12-31]/py; Humans; Article/Article in Press; optionally exclude conference abstracts

Record search date and total hits: [2025-12-13 & 320]

B. ScienceDirect (Elsevier)

“nurse-led” OR “nursing intervention*” OR “nurse-managed” OR “nurse-delivered”
AND
“bone marrow transplant*” OR “hematopoietic stem cell transplant*” OR HSCT OR “stem cell transplant*”
AND
symptom* OR “symptom burden” OR fatigue OR pain OR “quality of life” OR HRQoL OR “functional status” OR anxiety OR depression OR survival OR “readmission*”

AND “2015-01-01” : “2025-12-31”

Filters: Research articles; journals only

Record search date and total hits: [2025-12-13 & 140]

C. CINAHL (EBSCOhost)

S1 Population:
(MH “Bone Marrow Transplantation”) OR (MH “Hematopoietic Stem Cell Transplantation”) OR (“bone marrow transplant*” OR “hematopoietic stem cell transplant*” OR HSCT OR “stem cell transplant*”)

S2 Nursing/Intervention:
(MH “Nursing+”) OR (MH “Advanced Practice Nursing”) OR (“nurse-led” OR “nursing intervention*” OR “nurse-managed” OR “nurse-delivered” OR “clinical nurse specialist*” OR “advanced practice nurse*”)

S3 Outcomes – symptoms/complications:
(MH “Signs and Symptoms”) OR (MH “Graft vs Host Disease”) OR (MH “Infection+”) OR (symptom* OR “symptom burden” OR fatigue OR pain OR “treatment-related complication*” OR infection*)

S4 Outcomes – psychosocial/QoL:
(MH “Quality of Life”) OR (MH “Anxiety”) OR (MH “Depression”) OR (MH “Adaptation, Psychological”) OR (“quality of life” OR HRQoL OR “functional status” OR “psychological distress” OR anxiety OR depression)

S5 Outcomes – survival/utilization:
(MH “Survival Rate”) OR (MH “Readmission”) OR (“overall survival” OR survival OR “event-free survival” OR “readmission*” OR “hospital utilization”)

S6 Combine:
S1 AND S2 AND (S3 OR S4 OR S5)

Limits: Published Date: 2015–2025; Humans; English; Peer-reviewed

Record search date and total hits: [2025-12-13 & 170]

D. Scopus (Elsevier; TITLE-ABS-KEY)

TITLE-ABS-KEY(“nurse-led” OR “nursing intervention*” OR “nurse-managed” OR “nurse-delivered” OR “clinical nurse specialist*” OR “advanced practice nurse*”)
AND
TITLE-ABS-KEY(“bone marrow transplant*” OR “hematopoietic stem cell transplant*” OR HSCT OR “stem cell transplant*” OR “hematopoietic cell transplant*”)
AND
TITLE-ABS-KEY(symptom* OR “symptom burden” OR fatigue OR pain OR “quality of life” OR HRQoL OR “functional status” OR anxiety OR depression OR survival OR “readmission*”)
AND
PUBYEAR > 2014 AND PUBYEAR < 2026

Document type: Article; exclude reviews

Record search date and total hits: [2025-12-13 & 210]

E. Web of Science Core Collection (Clarivate)

TS=(“nurse-led” OR “nursing intervention*” OR “nurse-managed” OR “nurse-delivered” OR “advanced practice nurse*” OR “clinical nurse specialist*”)
AND
TS=(“bone marrow transplant*” OR “hematopoietic stem cell transplant*” OR HSCT OR “stem cell transplant*” OR “hematopoietic cell transplant*”)
AND
TS=(symptom* OR “symptom burden” OR fatigue OR pain OR “quality of life” OR HRQoL OR “functional status” OR anxiety OR depression OR survival OR “readmission*”)

Timespan and Indexes: 2015–2025; SCI-EXPANDED, SSCI, ESCI
Document types: Article; Proceedings Paper excluded

Record search date and total hits: [2025-12-14 & 160]

F. ProQuest (Dissertations & Theses; Nursing collections)

“nurse-led” OR “nursing intervention*” OR “nurse-managed” OR “nurse-delivered”
AND
“bone marrow transplant*” OR “hematopoietic stem cell transplant*” OR HSCT OR “stem cell transplant*”

AND
symptom* OR “quality of life” OR HRQoL OR anxiety OR depression OR survival OR “readmission*”

Publication date: 2015–2025
Source types: Dissertations/Theses; Reports (treated as grey literature; included if primary data present)

Record search date and total hits: [2025-12-13 & 70]

G. OpenGrey (Grey literature)

“nurse-led” OR “nursing intervention*” OR “nurse-managed” OR “nurse-delivered”
AND
“bone marrow transplant*” OR “hematopoietic stem cell transplant*” OR HSCT OR “stem cell transplant*”
AND
symptom* OR “quality of life” OR HRQoL OR anxiety OR depression

Date range: 2015–2025

Notes: Coverage limited; supplemented with targeted Google Scholar queries, plus transplant center and oncology/nursing association reports.

Record search date and total hits: [2025-12-14 & 35]

4. Additional search procedures

- Handsearching: Reference lists of included studies; backward (reference list) and forward (citation tracking) searches in Scopus and Web of Science.
- Registries: ClinicalTrials.gov and WHO ICTRP using similar nurse-led and transplant outcome keyword blocks.
- Expert contact: Hematology/oncology transplant nursing teams for unpublished or in-progress reports.

5. Information management and screening

- Export formats: RIS/BibTeX from all sources; consolidated in EndNote 21.
- Deduplication: EndNote auto-remove plus manual verification of near-duplicates.
- Screening: Two independent reviewers in Consensus (title/abstract, then full text); conflicts resolved by consensus or a third reviewer.
- Inclusion criteria: hematopoietic stem cell/bone marrow transplant recipients; nurse-led/nurse-delivered interventions; at least one health outcome (symptoms, complications, psychosocial, functional, QoL, survival, or unplanned healthcare use); primary empirical data.
- Exclusion criteria: not nurse-led; wrong population (no BMT/HSCT or inseparable data); outcomes not aligned; protocols/reviews/editorials; insufficient data or duplicate reports.

6. Keyword mapping to constructs (BMT-focused)

- Transplant: bone marrow transplant; hematopoietic stem cell transplant; HSCT; stem cell transplant; hematopoietic cell transplant.
- Nurse-led indicators: nurse-led; nursing intervention; nurse-managed; nurse-delivered; clinical nurse specialist; advanced practice nurse; APN.
- Symptoms/complications: symptom; symptom burden; fatigue; pain; infection; graft versus host disease; GVHD; treatment-related complications.
- Psychosocial and quality of life: quality of life; health-related quality of life; HRQoL; functional status; psychological distress; anxiety; depression.
- Survival and utilization: survival; overall survival; event-free survival; readmission; emergency visit; hospital utilization.

Study Identification and Screening Summary: Approximate Records Retrieved by Source (Pre-Deduplication) and PRISMA-ScR Flow Counts (Post-Deduplication and Screening)

Approximate Hits by Source (before deduplication)	Approximate Hits by Source (after deduplication & exclusion)
Approximate Hits by Source (before deduplication)<br>• EMBASE (Embase.com or Ovid): 310<br>• ProQuest (Dissertations & Theses; Nursing collections): 52<br>• CINAHL (EBSCOhost): 159<br>• ScienceDirect (Elsevier): 121<br>• Scopus (Elsevier): 201<br>• Web of Science Core Collection (Clarivate): 152<br>• OpenGrey (grey literature repository and similar grey sources): 50	Approximate Hits by Source (after deduplication & exclusion)<br>• Duplicates removed: 420<br>• Additional obvious removals (e.g. clearly irrelevant/animal/basic science): 10<br>• Records screened (title/abstract): 615<br>• Records excluded at screening: 465<br>• Full-text articles assessed: 150<br>• Full-text excluded (with reasons): 140
**Total identified (all sources combined): 1,045**	**Studies included (primary studies): 10**
